# Efficient Generation of Mice with Consistent Transgene Expression by FEEST

**DOI:** 10.1038/srep16284

**Published:** 2015-11-17

**Authors:** Lei Gao, Yonghua Jiang, Libing Mu, Yanbin Liu, Fengchao Wang, Peng Wang, Aiqun Zhang, Nan Tang, Ting Chen, Minmin Luo, Lei Yu, Shaorong Gao, Liang Chen

**Affiliations:** 1 College of Life Sciences, Beijing Normal University, Beijing, 100875, China; 2 National Institute of Biological Sciences, Beijing, Beijing 102206, China; 3 School of Life Science, Tsinghua University, Beijing 100084, China; 4 National Institute of Biological Sciences, Collaborative Innovation Center for Cancer Medicine, Beijing, 102206, China; 5 Beijing Ditan Hospital, Beijing 100015, China; 6 General Hospital of People’s Liberation Army, Beijing 100853, China; 7 Beijing Tongren Hospital, Capital Medical University, Beijing 100730, China; 8 Tongji University, Shanghai, 200092, China

## Abstract

Transgenic mouse models are widely used in biomedical research; however, current techniques for producing transgenic mice are limited due to the unpredictable nature of transgene expression. Here, we report a novel, highly efficient technique for the generation of transgenic mice with single-copy integration of the transgene and guaranteed expression of the gene-of-interest (GOI). We refer to this technique as functionally enriched ES cell transgenics, or FEEST. ES cells harboring an inducible Cre gene enabled the efficient selection of transgenic ES cell clones using hygromycin before Cre-mediated recombination. Expression of the GOI was confirmed by assaying for the GFP after Cre recombination. As a proof-of-principle, we produced a transgenic mouse line containing Cre-activatable tTA (cl-tTA6). This tTA mouse model was able to induce tumor formation when crossed with a transgenic mouse line containing a doxycycline-inducible oncogene. We also showed that the cl-tTA6 mouse is a valuable tool for faithfully recapitulating the clinical course of tumor development. We showed that FEEST can be easily adapted for other genes by preparing a transgenic mouse model of conditionally activatable EGFR L858R. Thus, FEEST is a technique with the potential to generate transgenic mouse models at a genome-wide scale.

Research in the post-genomic era requires the functional characterization of proteins^1^,^2^. The mouse is an attractive and popular model organism for the *in vivo* study of protein function, and two recent works have reported the direct^3^ and conditional^4^ knockout of protein coding genes at a genome-wide scale. However, the approaches described in these two reports provide loss-of-function mouse models. It is equally important to construct a gain-of-function platform through transgenic engineering.

The traditional method of generating transgenic mice is based on the microinjection of a construct into the pronucleus of a fertilized egg^5^,^6^. This technique has facilitated much of our understanding of the *in vivo* functions of cis-regulatory elements and proteins. However, this technique has several disadvantages: 1) the characterization of founder lines is labor intensive; 2) the characterization of the transgene insertion is performed in the mouse, which is time-consuming in many cases and can be limited by the physiological development of the mouse; 3) only a limited number of founder lines are characterized; 4) an adequate expression level of the transgene is not guaranteed; and 5) multiple copies of the transgene can be integrated into the mouse genome in a head-to-tail fashion at a single site, potentially resulting in silencing of the transgene^7^. Recently, several modified techniques have been introduced. For example, transposon-based microinjection has been shown to produce single-copy transgenic founder lines^8^. Methods for integrating the transgene at a target site, such as Rosa26^9^ or ColA1^10^, have also been developed. Despite these improvements, no current transgenic protocol can guarantee the expression of a transgene in the resultant founder lines. We therefore propose an efficient technique for generating transgenic mice that addresses the problems described above.

Mouse models expressing transgenes in a spatially and temporally-controlled fashion have been utilized to recapitulate many disease conditions. For example, a mouse model of lung cancer requires that a transgene (usually a human oncogene) be expressed in the lung epithelium of the adult mouse. A doxycycline-inducible system is commonly used to produce gene expression that can be regulated^11^. Thus, many organ-specific rtTA (reverse tetracycline transcriptional activator) driver mouse lines have been developed by researchers for the study of various organs. Alternatively, a universally applicable driver mouse line for the organ-specific expression of a Tet-controlled transgene might serve the same purpose if it were available.

Here, we report a highly efficient technique for generating transgenic mice, FEEST (functionally enriched ES cell transgenics), which guarantees the expression of the transgene. We made a CAG-lsl-tTA transgenic mouse model (referred to as cl-tTA) to demonstrate the application of this method. The predicted function of the tTA gene in cl-tTA mice was confirmed by driving lung cancer tumorigenesis when crossed with TetO-KrasG12C mice. cl-tTA mice are a valuable tool for faithfully recapitulating the clinical course of tumor development. We also generated a CAG-lsl-EGFR L858R transgenic mouse line to demonstrate that this technique can be adapted to other genes.

## Results

### Principle of the FEEST technique

**Figure 1:**
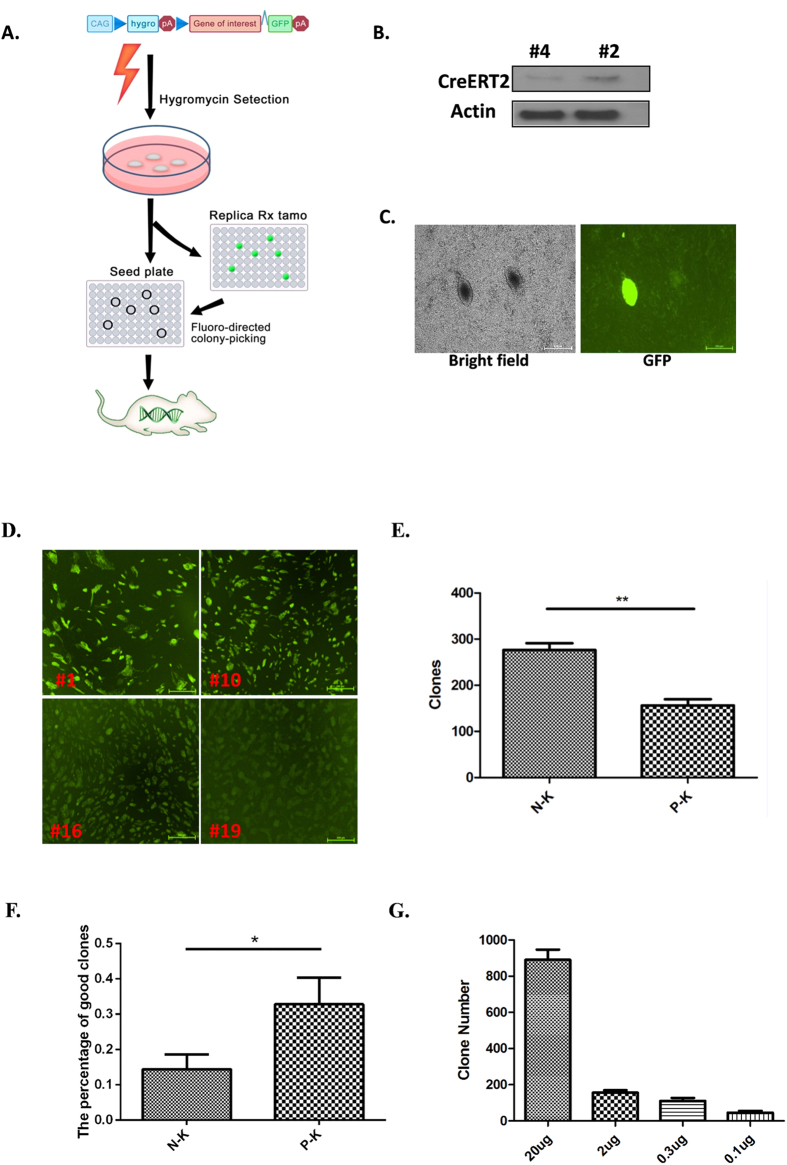
Establishment of FEEST. (**A**) Schematic of FEEST. (The figure is made by Mr. Ning Yang for publication under Open Access license). (**B**) Establishment of rosa26-CreERT2 ES cells. Lysates were analyzed by immunoblotting with anti-Cre and anti-β-actin antibodies, and gels were run under the same experimental conditions. (**C**) Inducible ES cells are identified. (**E**) Pseudo-Kozak sequence interference to enrich good clones. N-K: no Kozak sequence; P-K: pseudo-Kozak sequence. (**F**) The percentage of good clones doubled in the P-K cohort. (**G**) Titration of the DNA used in electroporation.

A schematic of the FEEST technique is provided in Fig. 1A. We used a strong promoter, CAG^12^, for universal transgene expression in mouse organs. A floxed hygromycin resistance selection marker followed by a polyA signal was placed between the CAG promoter and the gene of interest (GOI). To monitor the expression of the GOI, it was tagged with an IRES-GFP, such that the GOI and GFP would be expressed simultaneously but would not be fused. We refer to this construct as “CAG-lhl-GOI-GFP.” This strategy enables the expression of the hygromycin resistance gene in successful transfectants prior to Cre-mediated recombination. Then, upon the delivery of Cre activity, the GOI and GFP are expressed.

We first derived ES cell lines from embryos of rosa-creERT2 transgenic mice^13^. A total of 10 clones were established. Clones #2 and #4 (Fig. 1B present Western blots showing creERT2 expression) were confirmed to have normal karyotypes (data not shown) and totipotency (by confirming all 3 germ-layers in teratomas derived from each clone; Supplementary Fig. 1A). We also confirmed that both clones were capable of efficiently integrating into chimeric mice (Supplementary Fig. 1B) and that these mice gave birth to rosa-creERT2 F1 offspring at a Mendelian ratio.

To test this system, we generated a construct featuring CAG-lhl-GFP and electroporated it into CreERT2-expressing ES cells and selected the successful transfectants with hygromycin. Every surviving clone was divided equally and transferred to a seed plate and a replica plate. We screened the clones on the replica plate by applying 2 μg/ml of tamoxifen. We then checked the GFP signal 48 hours after tamoxifen treatment. Interestingly, the majority of the hygromycin-resistant clones were GFP negative, with GFP-positive clones accounting for only approximately 15% of the total clones (Fig. 1C). We found that the intensity of the GFP signal varied among the clones, and we scored the clones as strong, intermediate and weak according to the intensity of the GFP signal (Fig. 1D). We refer to strongly positive clones as “good clones” because the GOI was not expressed prior to delivery of Cre and was strongly expressed after Cre-mediated recombination. Good clones were found to comprise 30% of GFP positive population.

Overall, good clones were rare: only 15% of all hygromycin-resistant clones were GFP positive, and just 30% of all GFP-positive clones were strong GFP expressers. We attempted to increase the percentage of good clones through hygromycin selection. The Kozak sequence can recruit ribosomes for translation, and sequences containing a downstream frame-shifted ATG have a low possibility of being translated^14^. We placed a frame-shifted Kozak sequence (labeled as pseudo-Kozak, P-K) upstream of the hygromycin resistance gene to down-regulate the level of the hygromycin resistance protein. In clones containing a compromised CAG promoter, expression of the hygromycin resistance gene is critically low, such that the clone does not survive hygromycin selection. Only those clones whose CAG promoter is integrated into an environment suitable for full activation can compensate for P-K interference and survive hygromycin selection. We assayed the activity of the expressed hygromycin resistance gene by counting the resistant clones. As expected, the P-K-modified construct resulted in approximately half the number of resistant clones compared to the non-Kozak-modified construct (N-K; Fig. 1E), resulting in a nearly doubled percentage of good clones in the P-K group compared to the N-K group (Fig. 1F).

The presence of a single copy of a transgene is ideal for generating transgenic mice, particularly in the Cre-loxP activated system, because the presence of multiple LoxP sites complicates Cre-mediated recombination. To generate single-copy transgenic ES cells, we titrated the transgene DNA used for electroporation and assayed the number of transgene copies present in the hygromycin-resistant clones. We found that reducing the amount of DNA used for electroporation led to a lower copy number of the transgene in ES cell clones and that a single copy of the transgene was detected in each of the 80 clones assayed when 0.3 μg of DNA was used for electroporation. We also found that electroporation with 0.3 μg of DNA resulted in approximately 100 hygromycin-resistant clones on a single 10-cm plate (Fig. 1G). For subsequent experiments, we therefore used 0.3 μg DNA for electroporation.

Under these conditions, we routinely identified between 5 and 10 “good” clones from a single 10-cm plate. We named this technique “functionally enriched ES cell transgenics,” or “FEEST.”

Of note, in traditional ES cell engineering, an entire 96-well plate containing both good and bad clones is frozen and screened at a later date to identify the clones that are suitable for generating mice. With FEEST, the good clones are identified 48 hours following transfer from the selection plate and are immediately ready for injection. Thus, FEEST significantly reduces the amount of time and labor required to identify good ES cell clones.

### Conditional tTA transgenic mice established by FEEST

The Tet-inducible system is a powerful tool for controlling transgene expression. The currently available universal (r)tTA mouse lines are driven either by the rosa26^15^ or the CAG^16^ promoter, or they are conditionally driven via rosa26^17^,^18^. The availability of a conditional tTA transgene driven by the CAG promoter will be of importance for the study of organs that currently lack a specific (r)tTA transgenic line for the overexpression of a Tet-controlled transgene. We therefore demonstrated that FEEST is a powerful approach for the generation of conditional tTA transgenic mice.

**Figure 2:**
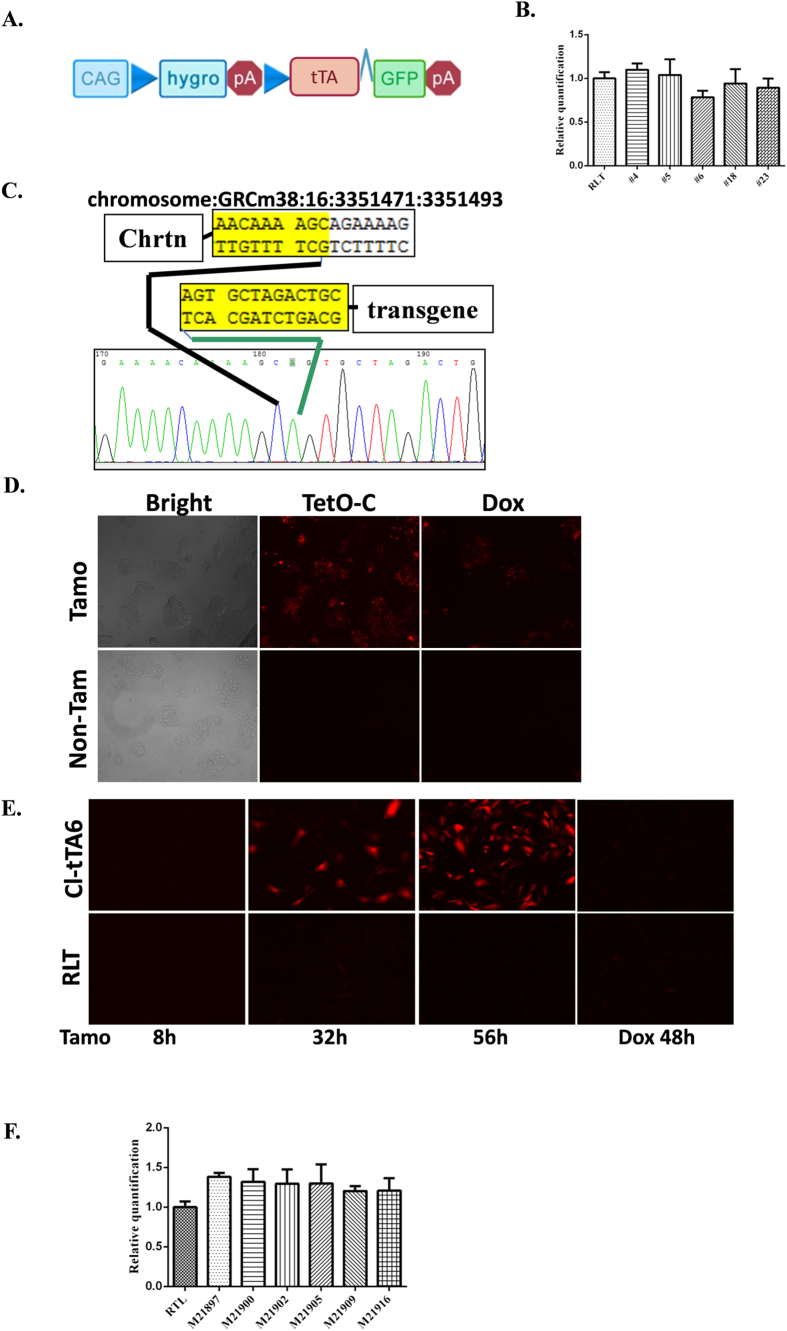
Establishment of CAG-lsl-tTA ES cells. (**A**) The schematic of cl-tTA. tTA: tetracycline transcriptional activator. (**B**) cl-tTA transgene copy number determination. RLT: genomic DNA from rosa-lsl-tTA. (**C**) The integration site of the transgene. Chrtn: chromatin DNA. (**D**) Tightly regulated tTA function in cl-tTA6 ES cells. Upper row: tamoxifen treated. Lower row: non-tamoxifen treated. (**E**) Higher levels of tTA function seen in cl-tTA transgenic mice than in rosa-lsl-tTA mice. (**F**) transgene copy number in offspring of the cl-tTA6 founder.

The construct of CAG-lhl-tTA-GFP was shown in Fig. 2A. We next electroporated the CAG-lhl-tTA-GFP construct into clone #4. Six good clones were identified by the strong GFP signal induced by tamoxifen treatment. We also confirmed that they harbored only a single copy of the transgene (Fig. 2B). Among the good clones, we randomly chose CAG-lhl-tTA-GFP #6 (cl-tTA6) for further analysis. We further confirmed the integration site of the transgene by TAIL-PCR. Indeed, we found that the transgene was integrated at bp 3351493 of chromosome 16 (Fig. 2C), providing additional proof that cl-tTA6 harbors a single copy of the transgene.

We identified good clones based on the GFP signal as a surrogate of tTA expression. However, it remains to be determined whether the tTA protein is expressed and functional in these clones. To test the tTA function, we delivered the Tet-mCherry gene using a retrovirus. We found that while the transgenic ES cells did not exhibit mCherry fluorescence, tamoxifen treatment led to a strong mCherry signal (Fig. 2D). This result demonstrated that the tTA protein could be induced by Cre recombination. Because tTA drives tet-controlled genes in a tet-off manner, we further tested whether doxycycline treatment could quench mCherry signal. Indeed, doxycycline administration led to the rapid regression of the mCherry signal. cl-tTA6 transgenic ES cells, therefore, exhibited tight control over the expression of fully functional tTA (Fig. 2D).

We next generated transgenic mice containing cl-tTA6. We found that more than 90% of the skin of chimeric mice was derived from cl-tTA6 ES cells (data not shown). These chimeric mice readily transferred the cl-tTA transgene allele to the F1 offspring. To test the function of cl-tTA in these mice, we isolated ear fibroblasts. As a point of comparison for tTA protein function, we isolated ear fibroblasts from rosa-lsl-tTA/rosa-CreERT2 bitransgenic mice^18^. Tet-mCherry retroviruses were delivered to visually monitor the function of the tTA protein. Interestingly, we found that while the rosa-lsl-tTA mice were positive for the mCherry signal, the CAG-lhl-tTA mice showed a stronger signal in response to tamoxifen treatment. The mCherry signal in both cell types was efficiently eliminated by doxycycline treatment for 48 hours (Fig. 2E).

We confirmed the copy number of the transgene in the F1 offspring and found that these mice harbored a single copy of the transgene (Fig. 2F).

### cl-tTA activates tet-controlled transgenes *in vivo*

The main purpose of cl-tTA transgenic mice is to drive the expression of Tet-controlled genes in an organ-specific manner, which recapitulates the pathogenesis of many abnormalities, such as tumorigenesis. We therefore tested whether cl-tTA6 mice would be useful for cancer research.

**Figure 3:**
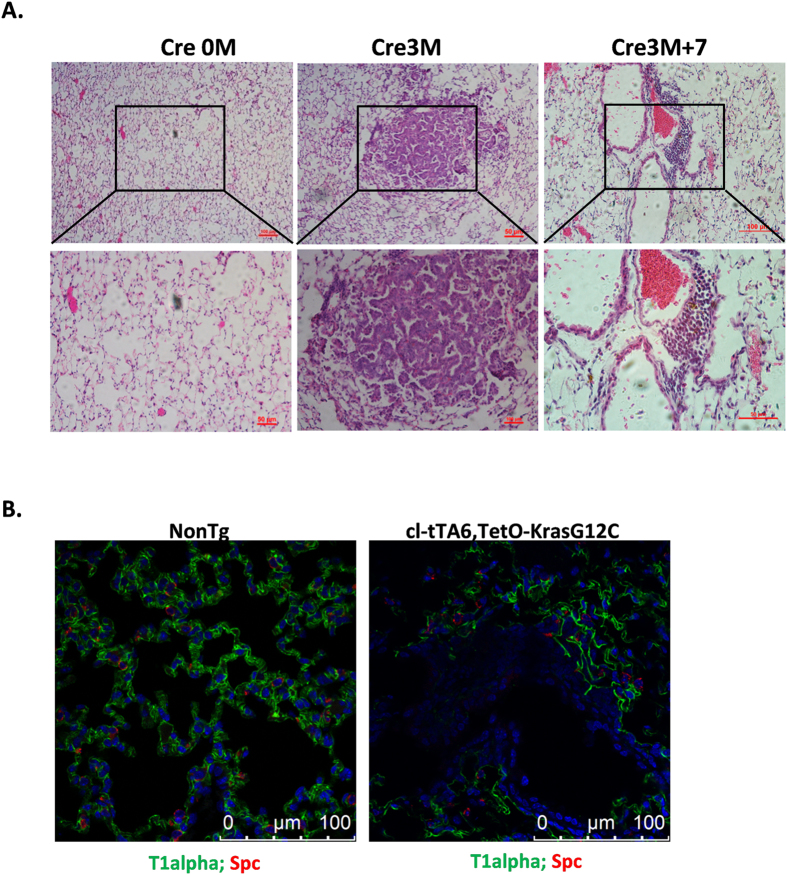
Controllable tumorigenesis in cl-tTA6 mice. (**A**) Lung cancer development in cl-tTA6/TetO-KrasG12C bitransgenic mice. Cre 0 M: untreated, Cre 3 M: 3 months post adeno-Cre virus treatment, Cre 3M + 7: doxycycline diet for 7 days after Cre 3 M. (**B**) Fluorescent immunohistochemical characterization of lung cancer. NonTg: non transgenic mice.

Kras G12C is an oncogenic mutation that is frequently found in lung cancer patients and is responsible for tumorigenesis^19^,^20^. We generated a TetO-KrasG12C transgenic mouse line and crossed it with the cl-tTA6 line to generate bitransgenic mice. These TetO-KrasG12C/cl-tTA6 bitransgenic mice were viable and are tumor free for the duration of their lifespan (Fig. 3A, left panel). We nasally administered the Cre virus into a cohort of bitransgenic mice at 1 month old and found that these mice developed poorly differentiated lung adenocarcinoma at 3 months post-infection (Fig. 3A, middle panel), consistent with previous reports^21^. Earlier work has shown that discontinuing the expression of mutant Kras leads to regression of the tumor. We therefore fed the tumor-bearing mice a diet containing doxycycline for 7 days. Interestingly, tumor regression was observed in these mice, as indicated by the increased intra-tumoral spaces and thickened alveolar walls, suggesting remodeling of the area originally occupied by the tumor (Fig. 3A, right panel). These results are consistent with an earlier report that mutant Kras-driven lung cancers require the continuous expression of the oncogene for maintenance.

To further validate that cl-tTA6/TetO-KrasG12C drives tumor formation in a manner that recapitulates tumorigenesis in patients, we assayed the expression of lung cancer markers using immunofluorescence microscopy (IFM). We found that the tumors were negative for T1α^22^ and SPC^23^ (markers of AEC II or AEC I cells, respectively) (Fig. 3B).

### cl-tTA mice is useful to faithfully recapitulate the clinical course of tumor development in patients

The clinical course of tumor development is a process in which a somatic cell that contains a mutation capable of initiating tumorigenesis accumulates additional mutations, until the tumor gains the capability to metastasize to distal organs. Unfortunately, current doxycycline-inducible mouse models drive oncogene expression with a specific promoter, resulting in expression of the oncogene in all of the promoter-active cells. We wanted to confirm that our cl-tTA transgenic line offers an alternative for the faithful recapitulation of tumor development by testing it on skin tumors.

**Figure 4:**
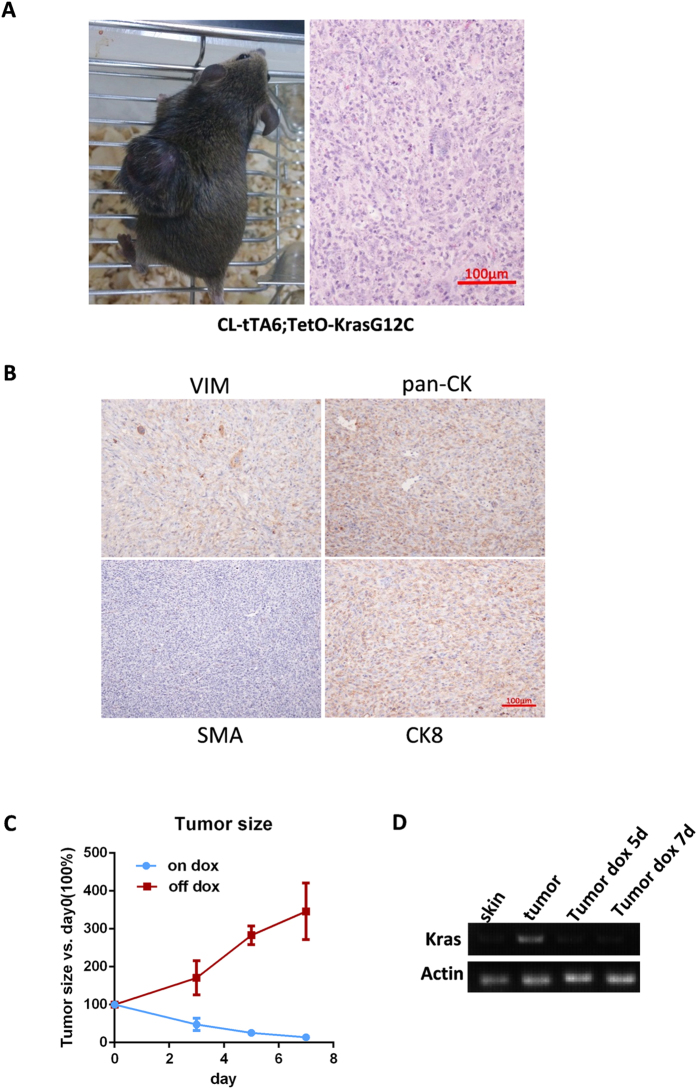
cl-tTA6 for focal tumorigenesis. (**A**) Single tumor induced in cl-tTA/TetO-KrasG12C bitransgenic mice. Left panel: picture of the mice; right panel: histology of the skin tumor. (**B**) Cell component analysis of the tumor nodule. (**C**) Doxycycline treatment leads to regression of the tumor. (**D**) Controllable expression of KrasG12C.

Skin squamous cell carcinoma has traditionally been modeled in mice by DMBA/TPA treatment, which induces random Ras mutagenesis and promotes tumor initiation and progression. This approach is lengthy and can require between 2 and 6 months for a tumor to develop, depending on the mouse strain, with unpredictable patterns. However, transgenic overexpression of mutant Ras by the Keratin-14 or -5 promoter, which are expressed in all epithelial cells, leads to broad hyperproliferation and early death rather than physiologically relevant tumor formation. Our cl-tTA mice could provide a method for recapitulating the clinical course of tumorigenesis in a spatially and temporally controlled fashion. To this end, we spot-injected Cre-expressing virus into the dermis of cl-tTA6/TetO-KrasG12C mice. Interestingly, 45 days after infection, a single large tumor developed at the injection site, while other areas remained tumor-free (Fig. 4A). Pathological examination revealed the poorly differentiated cancer and that the tumor consisted mostly of large cohesive cells that displayed the pleomorphic or anaplastic features seen in sarcoma. All large cells had abundant cytoplasm and prominent nuclei. The immunohistochemical staining revealed that the tumor cells were not immunoreactive for actin or smooth muscle actin, but they were positive for pan-cytokeratin (AE1/AE3)^24^ and cytokeratin 8^25^, and they were focally positive for vimentin^26^ (Fig. 4B, negative control for all these IHCs were shown in supplementary Figure 2A). In immunofluorescent staining of frozen tumor sections, the tumor cells were shown to be positive for cytokeratin 14^27^ (upper panel, Supplementary Figure 2 B), which is indicative of the tumor’s epithelial origin. Endothelium cell (CD31 positive^28^) (middle panel, Supplementary Figure 2 B) and focal oil-red-O stains were also observed, which indicated the presence of lipid vacuoles in the cytoplasm (lower panel, Supplementary Figure 2B).

The expression of activated mutant Kras in the tumor initiated by Cre viral infection of cl-tTA6/TetO-KrasG12C bitransgenic mice can be turned off by doxycycline treatment, providing an excellent model to test whether the tumors are dependent on continuous expression of mutant Kras for maintenance. To this end, we transplanted tumors into nude mice and fed the mice diets with or without doxycycline once the tumor reached approximately 200 mm^3^. As expected, the doxycycline diet led to a rapid regression of the tumor xenografts, while the normal diet resulted in the xenografts doubling their volume every 3 days (Fig. 4C). In addition, we observed that the expression of the Kras transgene was rapidly quenched by doxycycline treatment (Fig. 4D).

### FEEST efficiently generates transgenic mouse lines harboring Cre activatable EGFR L858R

**Figure 5:**
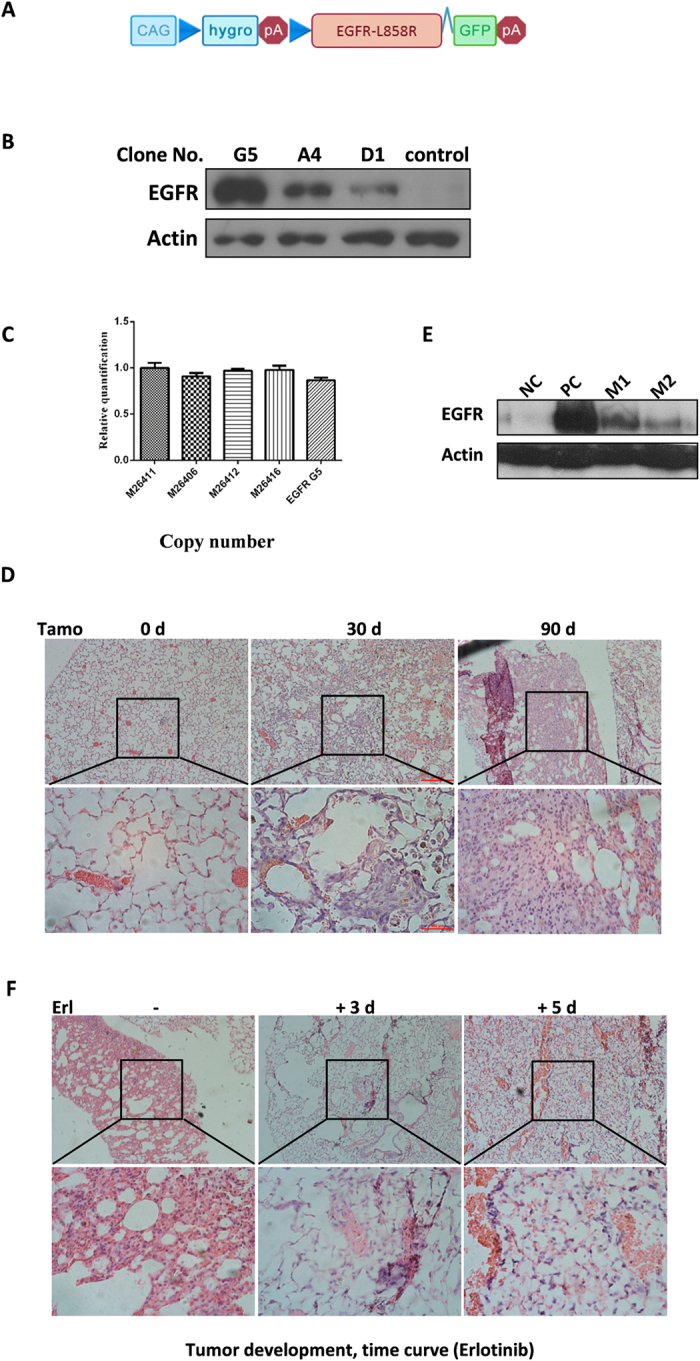
Characterization of cl-EGFR L858R transgenic mice. (**A**) The schematic of cl-EGFR L858R. (**B**) FEEST efficiently identified good clones. G5 represents a good clone. Lysates were analyzed by immunoblotting with anti-EGFR and anti-β-actin antibodies and gels were run under the same experimental conditions. (**C**) Offspring and ES cells harbor a single copy of the transgene. (**D**) Controllable tumorigenesis in cl-EGFR L858R mice. Tamo 0d, 30d, 90d: 0 day, 30 days, and 90 days post tamoxifen treatment. (**E**) Controllable expression of EGFR L858R. β-actin and EGFR were detected by Western blot and gels were run under the same experimental conditions. M1 and M2: 2 representative mice; NC (negative control) and PC (positive control): TetO-EGFR L858R/cc10rtTA mice treated without or with doxycycline diet. (**F**) EGFR L858R-driven lung cancers are sensitive to erlotinib treatment. (Erlotinib −,  +3d, +5d: erlotinib treated for 0 day, 3 days and 5 days respectively).

We have clearly demonstrated that FEEST is a highly efficient method for the generation of transgenic mouse models and that it guarantees transgene expression, as shown by our proof-of-principal demonstration with cl-tTA6. To prove that FEEST can be used to generate mice that are transgenic for other genes, we set out to generate transgenic mice for EGFR L858R, one of the mutant EGFRs that is most frequently found in lung cancer patients^29^. The schematic construct of cl-EGFR L858R was showed in Fig. 5A. Good ES clones capable of inducing a high level of EGFR upon tamoxifen treatment were efficiently identified by assaying the GFP signal, as confirmed by Western blot analysis (Fig. 5B). We verified that the EGFR transgene was integrated into the genome in a single-copy manner (Fig. 5C). Most importantly, the SPC-CreERT2/cl-L858R mice developed early lesions of hyperplasia in the lungs one month after tamoxifen treatment (Fig. 5D, middle panel). By three months post-treatment, the pathology revealed a heavy tumor burden of adenocarcinoma in the lungs (Fig. 5D, right panel). Western blot analysis confirmed the expression of the EGFR transgene (Fig. 5E). To verify that these tumors recapitulated tumors observed in patients, we treated the mice with erlotinib, an FDA-approved EGFR inhibitor widely used in the treatment of lung cancer. Interestingly, we were able to discern an effect of treatment between 3 and 5 days after erlotinib administration in mice, as reflected by the occurrence of areas of thickened alveolar wall (Fig. 5F).

## Discussion

We have established a highly efficient technique, termed FEEST, for generating transgenic mice. One main advantage of this technique is that the expression of the transgene is guaranteed. Using FEEST, we have established a CAG-driven conditional tTA transgenic mouse model that efficiently elicits tumor formation in an organ-specific manner and faithfully recapitulates the clinical course of tumor formation observed in patients. Using the conditionally activatable EGFR-L858R, we also demonstrated that FEEST can be used to make transgenic mouse models for other genes.

In addition to traditional pronuclear injection, other methods for generating transgenic mouse models are currently in widespread use, making *in vivo* characterization of protein function more convenient. Rosa26 is a popular choice for site-directed transgenics due to its ability to drive the expression of exogenous genes. However, inexpressible transgenes are inevitably identified for any locus, and this technical frustration has been reported in well-proven vectors (e.g., pcDNA3.1), which are occasionally unable to drive the expression of gene fragments in eukaryotic cells. The reason for these failures remains to be elucidated. Nevertheless, the ability of FEEST to screen transgenic ES cell clones by transgene expression ensures the expression of the transgene in the resulting mouse upon activation by Cre recombination.

Conditional mouse transgenes driven by the CAG promoter have been widely reported^30^,^31^,^32^,^33^,^34^,^35^,^36^. These transgenes are generated either by site-directed targeting in ES cells or by direct microinjection. Compared to other techniques, FEEST features a higher efficiency of single-copy transgene integration, and it guarantees the expression of the transgene.

While this manuscript was in preparation, CAG-lsl-rtTA mice were reported by Lowe et. al.^37^. In these mice, rtTA drives the expression of a Tet-controlled transgene in a tet-on manner. Considering the inability of doxycycline to penetrate the blood-brain and blood-testis barriers^15^, expression of the transgene will not be induced in these tissues, even after Cre activation. The CAG-lsl-tTA transgene line reported in the current study will be of particular interest to researchers studying those tissues. Indeed, we detected expression of a transgene (Eml4-Alk) in the brains of cl-tTA/Eml4-Alk bitransgenic mice 3 weeks after surgical delivery of adeno-Cre virus into the brain (Supplementary Figure 3).

Tissue-specific (r)tTA driver lines fail to faithfully recapitulate the clinical course of tumor development. In the clinic, only a limited number of cells in an organ expressed an oncogene, which in most cases, arose through mutation. These cells then developed into tumor cells and led to the formation of tumor nodules. This process generates a very limited number of tumors. In contrast, tissue-specific (r)tTA driver lines can drive tet-controlled transgene expression in all cells where the promoter is active. Thus, in the (r)tTA mouse model of melanoma, for example, all melanocytes have the potential to be transformed after the induction of oncogene expression. The CAG-lsl-tTA allele, on the contrary, can induce the expression of an oncogene in a limited number of cells at a single focus by spot delivery of a Cre virus. In our work, we induced one single nodule originating from the skin. The CAG-lsl-tTA mouse is therefore a valuable tool for faithfully recapitulating the clinical course of tumor development.

FEEST is highly efficient in the identification of good clones. Hygromycin-resistant clones are split into two plates: a seed plate and a replica plate. Tamoxifen treatment is applied to the replica plate on the day of transfer, and the GFP signal is readily detectable 48 hours after seeding - well before clones in the seed plate are transferred to a 24-well plate for amplification. FEEST therefore bypasses the freezing down of a 96-well plate containing many uncharacterized clones. Instead, it allows the amplification of good clones for downstream steps at a very early stage, and it thus saves labor and resources.

Of course, researchers should be aware of some aspects of FEEST, which could potentially limit its application. FEEST screens ES cell clones by checking the expression level of the transgene. IRES-GFP element is built in with transgene, which could be an issue for lineage tracing study dependent on fluorescent protein expression. The transgene fragment in FEEST generated ES cell clones is randomly integrated into host genome. We suggest researchers to characterize multiple founder lines to make sure that the phenotype is not due to the positional effect. Manipulation of ES cells may limit its acceptance to some labs. Lastly, researchers may need to breed out the rosa-CreERT2 allele in most of the cases if the transgene needs to be expressed in a specific organ.

Given its high efficiency and the unique advantage of guaranteed inducible transgene expression, FEEST could be an important technique for generating transgenic mouse models at a genome-wide scale.

## Methods

### Ethics statement

All experimental protocols were approved by the Institutional Committee for Animal Care and Use, NIBS. All the methods were carried out in accordance with the approved guidelines.

### Mouse strains

All mice were housed in a pathogen-free environment at the National Institute of Biological Sciences, Beijing (NIBS). All mice were handled in strict accord with good animal practices, as defined by the Institutional Committee for Animal Care and Use, NIBS. The animal work was carried out according to the approved protocol.

To generate cl-tTA and cl-EGFR L858R transgenic mice, ES clones were injected with the ICR blastocysts, and chimeric embryos were implanted into pseudopregnant ICR recipients. Offspring were tail-genotyped and maintained in a dedicated pathogen-free facility in NIBS.

To generate the TetO-KrasG12C transgenic mice, Kras G12C-encoding cDNA fragments were cloned into pTRE-tight (CloneTech). TetO-KrasG12C-polyA fragments were then released by *Xho*I digestion and cloned into pCYL50^38^. The resulting pCYL50-Tet-Kras plasmid was co-injected into the pronuclei of fertilized eggs from an FVB background together with SB100× mRNA. The fertilized eggs were then implanted into pseudopregnant ICR recipients to derive transgenic mice. In our attempt to quantify the transgene copy number for cl-tTA6 transgenic mouse, we used genomic DNA samples of well characterized rosa-lsl-tTA which is single copy^18^ and determined based on the ratio of tTA between our mice and rosa-lsl-tTA mice through PCR analysis. To quantify the transgene copy number for cl-EGFR L858R, we take ES cell clone cl-tTA6 as single copy control to quantify the GFP fragment since they all have IRES-GFP fragment. The following primers were used for real-time PCR analysis. PCR condition and primers were listed in Section of “Semi-quantitative reverse transcription PCR and real-time PCR”.

### Plasmids and transfection

The plasmids used to produce the CAG-loxp-hygromycin-stop-loxp-GOI-IRES-GFP (CAG-lhl-GOI-GFP) and related information are available upon request. The transgene fragments were electroporated into Rosa-CreERT2 ES cells (please see establishment and characterization for ES cells). Transfected ES cells were subjected to selection with 140 μg/ml of hygromycin for 5–7 days until the ES cells were ready for transfer.

### Ear fibroblast culturing

Mice were euthanized, and the left and right pinna were removed. Each pinna was soaked in 70% EtOH for 5 min and transferred to sterile 1× PBS containing kanamycin (100 μg/ml). Collagenase (4 mg/ml) and protease (4 mg/ml) were added and the pinna were minced in one well of a 24-well plate. The digested tissues were passed through a cell strainer and pelleted by centrifugation at 1,020 *g* for 5 min. The cells were then resuspended and cultured in a 24-well plate.

### Semi-quantitative reverse transcription PCR and real-time PCR

Total RNA was isolated with TRIzol reagent (Invitrogen, Carlsbad, CA) following the manufacturer’s instructions. First strand cDNA was synthesized and reverse transcription was performed using the RevertAid™ First Strand cDNA Synthesis Kit (Thermo Scientific, Waltham, MA02451, USA). Real-time PCR was performed with the SYBR Green PCR Master Mix (Applied Biosystems) on an ABI PRISM 7500 Sequence Detection System. The following primers were used: GFP, 5′-GGTGAACTTCAAGATCCGCC-3′ and 5′-CTTGTACAGCTCGTCCATGC-3′; cl-tTA, 5′-GAAAATCAGCTCGCGTTCCT-3′ and 5′-GGGGCATAGAATCGGTGGTA-3′; and Mouse beta-Actin, 5′- ACAGCTTCTCTTTGATGTCACGC-3′ and 5′-TGTGATGGACTCCGGAGACGG-3′. The PCR thermal-cycling conditions were used as below: 95 °C for 10 min and 40 cycles at 95 °C for 3 s, at 60 °C for 30 s, and at 60 °C for 1 min.

### Western blotting

ES cells were maintained in Knockout DMEM supplemented with Lif, 15% knockout serum replacement serum, 2 mM L-glutamine, 1% nonessential amino acid, 0.1 mM β-mercaptoethanol, 1000 U/mL mouse LIF, 1 μM MEK inhibitor, and 3 μM GSK3β inhibitor. The cells were lysed in RIPA buffer (Beyotime, P0013E) supplemented with protease and phosphatase inhibitors (Roche). Western blotting was performed using standard methods. The blots were probed with the following antibodies: Human EGFR (Abcam, ab32077); Cre (Abcam, ab24607); actin (Sigma, A5316).

### Identification of transgene insertion sites

Tail-PCRs were performed as reported^39^. To identify the insertion site of the cl-tTA transgene, we designed the primers 5′-GAGCTAGTTCAAACCTTGGGAAAA-3′, 5′- CCATTTGCTTATCCTGCATCTCT-3′ and 5′-ACTTGAAGAAGGAAAAACAGGGG-3′.

### Skin tumor xenograft assay

An adeno-Cre virus was injected into the skin of cl-tTA;TetO-KrasG12C bitransgenic mice. One and a half months after injection, a large lump was observed at the site of injection and was confirmed by autopsy to be a tumor. The tumor was sectioned and injected into BALB/c nude mice (Vital River Laboratories). The size of the resultant tumor was monitored with digital calipers.

### H&E staining and immunofluorescence

Mouse lung tissue was embedded in paraffin, sectioned and stained with hematoxylin and eosin. Immunofluorescence staining was performed to detect T1alpha (Developmental Studies Hybridoma Bank clone 8.1.1), SPC (Millipore/Chemicon, AB3786), K14 (made at the Antibody Facility in NIBS), CD31 (BD Biosciences, 553369), vimentin (DAKO, IR63061), CK8 (Maixin, MAB-0167), Pan-CK (Thermo Scientific, MA5-13203), and SMA (Sigma, c6198).

### Statistical analysis

All quantified results were analyzed with GraphPad Prism software. Two-tailed paired *t*-tests were performed to evaluate significance. Bar graph data are presented as the mean ± SEM. The p values were denoted as *p < 0.05, **p < 0.01, and ***p < 0.001.

## Additional Information

**How to cite this article**: Gao, L. *et al.* Efficient Generation of Mice with Consistent Transgene Expression by FEEST. *Sci. Rep.* **5**, 16284; doi: 10.1038/srep16284 (2015).

## Supplementary Material

Supplementary Information
