# Author Correction: Efficient Generation of Mice with Consistent Transgene Expression by FEEST

**DOI:** 10.1038/s41598-018-32184-w

**Published:** 2018-10-16

**Authors:** Lei Gao, Yonghua Jiang, Libing Mu, Yanbin Liu, Fengchao Wang, Peng Wang, Aiqun Zhang, Nan Tang, Ting Chen, Minmin Luo, Lei Yu, Shaorong Gao, Liang Chen

**Affiliations:** 1 College of Life Sciences, Beijing Normal University, Beijing, 100875 China; 2 National Institute of Biological Sciences, Beijing, 102206 China; 3 School of Life Science, Tsinghua University, Beijing, 100084 China; 4 National Institute of Biological Sciences, Collaborative Innovation Center for Cancer Medicine, Beijing, 102206 China; 5 Beijing Ditan Hospital, Beijing, 100015 China; 6 General Hospital of People’s Liberation Army, Beijing, 100853 China; 7 Beijing Tongren Hospital, Capital Medical University, Beijing, 100730 China; 8 Tongji University, Shanghai, 200092 China

Correction to: *Scientific Reports* 10.1038/srep16284, published online 17 November 2015

The Acknowledgements section in this Article contains an error.

“The authors are grateful to Mr. Ning Yang in National Institute of Biological Sciences, Beijing, for making the picture in Figure 1A. The authors are grateful for Dr. Brigid L. M. Hogan for generously providing SPC-CreERT2 mice. This work was funded by Chinese Ministry of Science and Technology grant 2011CB812401, and the Beijing Municipal Government.”

should read:

“The authors are grateful to Mr. Ning Yang in National Institute of Biological Sciences, Beijing, for making the picture in Figure 1A. The authors are grateful for Dr. Brigid L. M. Hogan for generously providing SPC-CreERT2 mice. This work was funded by NSFC (81472606), and the Beijing Municipal Government.”

