# Supplementary material for: Efficient Generation of Mice with Consistent Transgene Expression by FEEST
Source: Sci Rep. 2015 Nov 17;5:16284. doi: 10.1038/srep16284 (PMC4648098; doi:10.1038/srep16284)
Supplement: Supplementary Information [file srep16284-s1.pdf]

Efficient Generation of Mice with Consistent Transgene Expression by FEEST

Lei Gao[1,2*], Yonghua Jiang[2*], Libing Mu[3*], Yanbin Liu[4], Fengchao Wang[2], Peng Wang[5], Aiqun Zhang[6], Nan Tang[2], Ting Chen[2], Minmin Luo[2], Lei Yu[7], Shaorong Gao[8#], Liang Chen[2,4#]

Running title: FEEST efficiently establishes transgenic mice to guarantee transgene expression

Supplementary Figure1: Pluripotency of Rosa-creERT2 ES cells. A. Hematoxylin and eosin staining of Roas-creERT2 ES cells-derived teratoma. Images of sections of teratomas, formed after transplantation of undifferentiated ESCs with their feeders and Matrigel into NOD/SCID mice. **B.** Chimeric mice derived from rosa-creERT2 #2 and #4 clones.

Supplementary Figure2: A. negative control of IHC in Figure 4B. A. Vimentin - negative uroepithelial carcinoma, Meningoma samples, which are negative for the markers were used as negative control for protein CK8, pan-CK and SMA. **B.** The expression of CK14, CD31 and lipid in the skin tumor cells. cl-tTA6; tetO-KrasG12C mice were spot-injected with cre expressing adeno-virus into skin dermis. Tumors developed were fluorescently stained with antibodies against K14 and CD31, with DAPI for nuclei (Negative control is performed with control IgG, followed by incubation of the respective secondary antibody used as in K14 or CD31 case). Oil Red O staining was stained to visualize lipid vacuoles in the cytoplasm.

Supplementary Figure3: The immunohistochemistry staining of Eml4-ALK in brains of Eml4-Alk;Cl-tTA6 transgenic mice. Mice were anesthetized with 5% isoflurane and placed into a stereotaxic frame. For all injections utilizing Bregma as a registration point, an incision was made to expose the skull and Bregma was visualized using a stereomicroscope. A hole was made overlying the targeted area by first thinning the skull using a fine drill burr until only a thin layer of bone remained. A microprobe and fine forceps were used to peel away this final layer of bone to reveal the brain surface. All mice received one unilateral injection into a single target region. Glass pipettes (inner tip diameter of 10 - 20 μm) loaded with virus were lowered to the desired depth from the Pial surface of the brain. Mice were quickly recovered after surgery and survived for 3 weeks prior to sacrifice.

Supplementary Figure 1

A

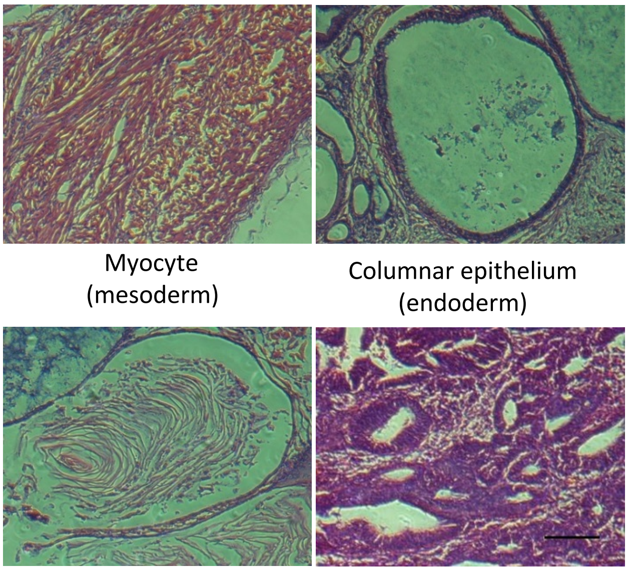

Myocyte (mesoderm)

Columnar epithelium (endoderm)

Keratinized epithelium (ectoderm)

Neurocyte (ectoderm)

B

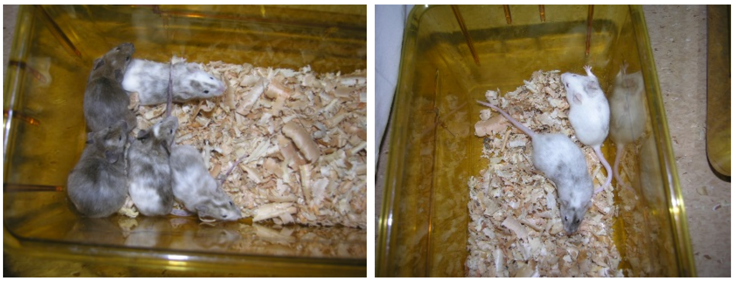

4# ES clone

2# ES clone

Supplementary Figure 2

A

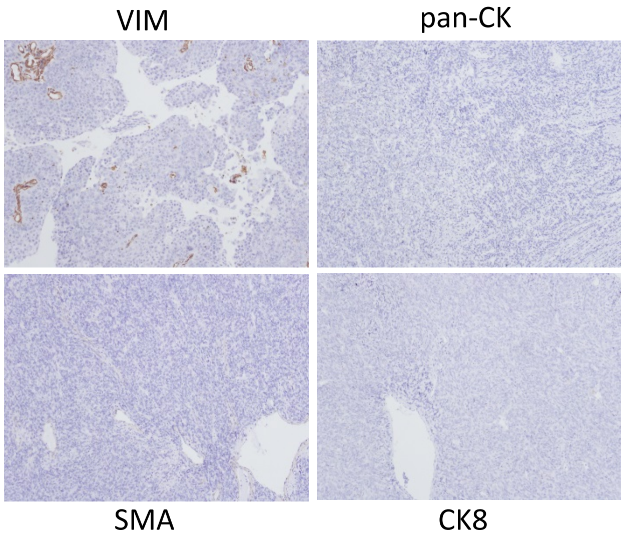

B

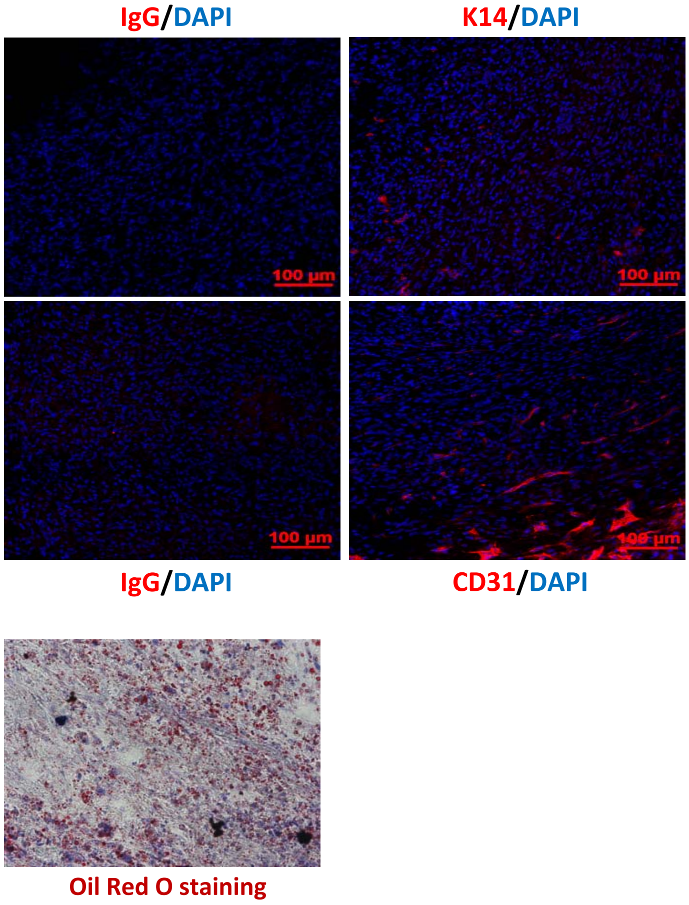

Supplementary Figure 3

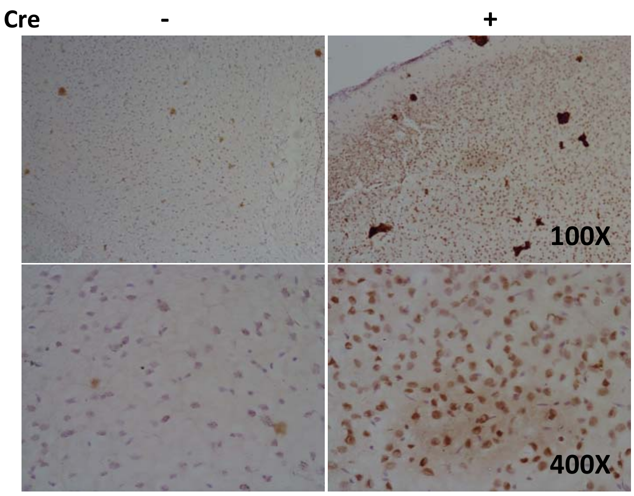

Eml4-Alk;cl-tTA6
